# A new regulator in the crossroads of oxidative stress resistance and virulence in *Candida glabrata*: The transcription factor CgTog1

**DOI:** 10.1080/21505594.2020.1839231

**Published:** 2020-10-31

**Authors:** Pedro Pais, Susana Vagueiro, Dalila Mil-Homens, Andreia I. Pimenta, Romeu Viana, Michiyo Okamoto, Hiroji Chibana, Arsénio M. Fialho, Miguel C. Teixeira

**Affiliations:** a Department of Bioengineering, Instituto Superior Técnico, Universidade de Lisboa, Lisbon, Portugal; b iBB - Institute for Bioengineering and Biosciences, Biological Sciences Research Group, Instituto Superior Técnico, Lisboa, Portugal; c Medical Mycology Research Center (MMRC), Chiba University, Chiba, Japan

**Keywords:** *Candida glabrata*, virulence, phagocytosis, CgTog1, oxidative stress, RNA-seq based transcriptomics

## Abstract

*Candida glabrata* is a prominent pathogenic yeast which exhibits a unique ability to survive the harsh environment of host immune cells. In this study, we describe the role of the transcription factor encoded by the gene *CAGL0F09229g*, here named CgTog1 after its *Saccharomyces cerevisiae* ortholog, as a new determinant of *C. glabrata* virulence. Interestingly, Tog1 is absent in the other clinically relevant *Candida* species (*C. albicans, C. parapsilosis, C. tropicalis, C. auris*), being exclusive to *C. glabrata*. CgTog1 was found to be required for oxidative stress resistance and for the modulation of reactive oxygen species inside *C. glabrata* cells. Also, CgTog1 was observed to be a nuclear protein, whose activity up-regulates the expression of 147 genes and represses 112 genes in *C. glabrata* cells exposed to H_2_O_2_, as revealed through RNA-seq-based transcriptomics analysis. Given the importance of oxidative stress response in the resistance to host immune cells, the effect of *CgTOG1* expression in yeast survival upon phagocytosis by *Galleria mellonella* hemocytes was evaluated, leading to the identification of CgTog1 as a determinant of yeast survival upon phagocytosis. Interestingly, CgTog1 targets include many whose expression changes in *C. glabrata* cells after engulfment by macrophages, including those involved in reprogrammed carbon metabolism, glyoxylate cycle and fatty acid degradation. In summary, CgTog1 is a new and specific regulator of virulence in *C. glabrata*, contributing to oxidative stress resistance and survival upon phagocytosis by host immune cells.

## Introduction

*Candida glabrata* is a prominent cause of invasive candidiasis, best known for its ability to resist azole antifungal therapy [1–5]. In contrast, the mechanisms allowing it to interact with the host and cause disease are more elusive, as typical virulence features displayed by the main pathogen *Candida albicans* are absent in *C. glabrata* [6].

Due to the absence of hyphae formation, secretion of hydrolases and host tissue damage, *C. glabrata* virulence has been associated with its ability to survive immune attack, rather than active immune escape or host tissue invasion (reviewed in [6,7]). Indeed, *C. glabrata* was found to be able to proliferate inside phagocytes, eventually leading to cell lysis due to intracellular fungal growth [8,9]. Genome-wide studies have granted a sneak peek into possible pathways that mediate *C. glabrata* immune survival [10]. The Oxidative Stress Response (OSR) pathway is regarded as a possible pathway for phagocyte survival, since oxidative stress caused by the production of Reactive Oxygen Species (ROS) is considered one of the main antifungal mechanisms applied by phagocytes [11,12].

The OSR in yeasts is centered on the activation of antioxidant systems based on the activity of catalase (*CTA1*), superoxide dismutases (*SOD1/2*), thioredoxin and glutathione pathways [13–16]. In yeasts, the transcription factors (TFs) Yeast-AP1 and Skn7 are the most well studied oxidative stress regulators [13,17]. Despite their well-defined role in mediating OSR, the actual role of this network (and the participation of each player) during phagocyte attack and virulence needs further clarification.

In this study, we identified the transcription factor CgTog1 as a regulator of oxidative stress resistance and phagocyte survival in *C. glabrata*. In a previous screening for zinc cluster transcription factor functions, CgTog1 had been shown to confer a slight increase in ketoconazole resistance, but no effect on hydrogen peroxide (H_2_O_2_) tolerance was detected under the chosen experimental conditions [18]. In previous studies, CgYap1 alone was not found to be required for *C. glabrata* macrophage survival nor virulence [13,19]. On the other hand, CgSkn7 was described to be involved in virulence, but not macrophage survival [13,20]. This study provides evidence for the new TF CgTog1 as a regulator required for OSR and phagocyte survival. In *Saccharomyces cerevisiae*, the Tog1 homolog has been implicated in oxidative stress tolerance, but it is best known for its role in regulation of fatty acid utilization genes and utilization of alternative carbon sources [21,22], a phenotype not observed in CgTog1.

Given the identification of *CgTOG1* as a new determinant of OSR in *C. glabrata*, its role in the transcriptome-wide response to H_2_O_2_ was assessed. The determination of CgTog1 targets enabled the identification of target genes sharing differential expression upon phagocytosis, which can be the basis for its role in hemocyte survival in a *Galleria mellonella* infection model. Additionally, this TF is absent in other *Candida* species (*C. albicans, C. parapsilosis, C. tropicalis, C. auris*), indicating a possible virulence regulatory network which is unique to *C. glabrata*.

## Results

### *CgTog1 confers resistance to oxidative stress, but is not involved in the utilization of alternative carbon sources in* C. glabrata

Upon phagocytosis, pathogens are faced with a multitude of antimicrobial stresses in the phagosome environment, including carbon limitation, which is often translated by the activation of pathways for the utilization of alternative carbon sources [6,10,23–26]. The TF Tog1 in the nonpathogenic yeast *S. cerevisiae* is required for the utilization of alternative carbon sources and resistance to oxidative stress [21]. Given the close phylogenetic relationship between *S. cerevisiae* and *C. glabrata*, we searched for a possible ortholog in the pathogenic yeast and assessed its functional conservation. Syntenic context visualized at YGOB [27] and protein homology searches show the TF encoded by *C. glabrata* ORF (open reading frame) *CAGL0F09229g* to be homologous to the *S. cerevisiae* Tog1 TF (26.46% identity). As such, this gene is henceforth designated *CgTOG1*.

A possible role for *CgTOG1* in the utilization of alternative carbon sources was investigated by comparing the growth of the wild type and the *Δcgtog1* mutant in the presence of lactate, glycerol or oleate. The deletion of *CgTOG1* does not result in growth impairment in the presence of non-fermentative carbon sources, as observed by spot assays (Figure 1a). Accordingly, *CgTOG1* overexpression via an expression plasmid did not provide any additional fitness for growth in the same conditions (Figure 1a). The attained results indicate that *CgTOG1* is not required for *C. glabrata* growth using alternative carbon sources, indicating that this TF does not share the same function as its *S. cerevisiae* counterpart. Another relevant antimicrobial mechanism applied in the phagosome is the production of ROS [6,10,14,28]. *C. glabrata* is notoriously more resistant to oxidative stress than other yeasts [15], which can be related with its ability to survive the phagosome environment. The participation of *CgTOG1* in oxidative stress resistance was investigated by spot assays, comparing the growth of the wild type and the *Δcgtog1* mutant in the presence of the oxidative stress inducers H_2_O_2_ and menadione. The *Δcgtog1* strain is more susceptible to H_2_O_2_ stress than the wild type strain (Figure 1b), indicating that it is required for *C. glabrata* tolerance to oxidative stress. Accordingly, its overexpression contributed to increased resistance against the same oxidative stress agent (Figure 1b). The overexpression of *CgTOG1* from the expression plasmid was validated by RT-PCR. Quantification of *CgTOG1* overexpression was found to be between 6 and 7-fold higher relative to the native *CgTOG1* expression (Supplementary Figure S1).

**Figure 1.**
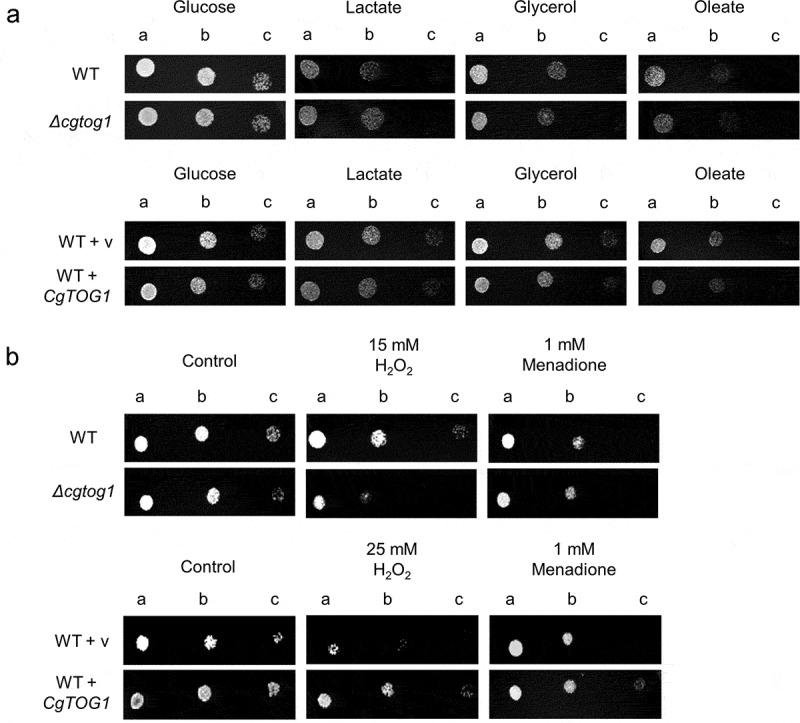
***CgTOG1* confers resistance to oxidative stress inducers but is not required for the utilization of alternative carbon sources**. (a) Comparison of spot growth assays of the KUE100 *C. glabrata* wild type and derived *Δcgtog1* deletion mutant, as well as the L5U1 *C. glabrata* wild type strain, harboring the pGREG576 cloning vector, or the pGREG576_MTI_*CgTOG1* expression plasmid, in the presence of glucose, lactate, glycerol or oleate as carbon sources. (b) Comparison of spot growth assays of the KUE100 *C. glabrata* wild type and derived *Δcgtog1* deletion mutant, as well as the L5U1 *C. glabrata* wild type strain, harboring the pGREG576 cloning vector, or the pGREG576_MTI_*CgTOG1* expression plasmid, in the presence of oxidative stress inducers H_2_O_2_ and menadione. (c) Comparison of spot growth assays of the KUE100 *C. glabrata* wild type and derived *Δcgtog1* deletion mutant, as well as the L5U1 *C. glabrata* wild type strain, harboring the pGREG576 cloning vector, or the pGREG576_MTI_*CgTOG1* expression plasmid, in the presence of distinct carbon sources and the oxidative stress inducer H_2_O_2_. (d) Comparison of spot growth assays of the KUE100::URA- *C. glabrata* wild type strain and the derived KUE100_*Δcgtog1*:URA- deletion mutant, harboring the pGREG576 cloning vector, or the pGREG576_MTI_*CgTOG1* expression plasmid, in the presence of the oxidative stress inducer H_2_O_2_. The inocula were prepared as described in the materials and methods section. Cell suspensions used to prepare the spots were 1:5 (b) and 1:25 (c) dilutions of the cell suspension used in (a). The displayed images are representative of at least three independent experiments

**Figure 1.**
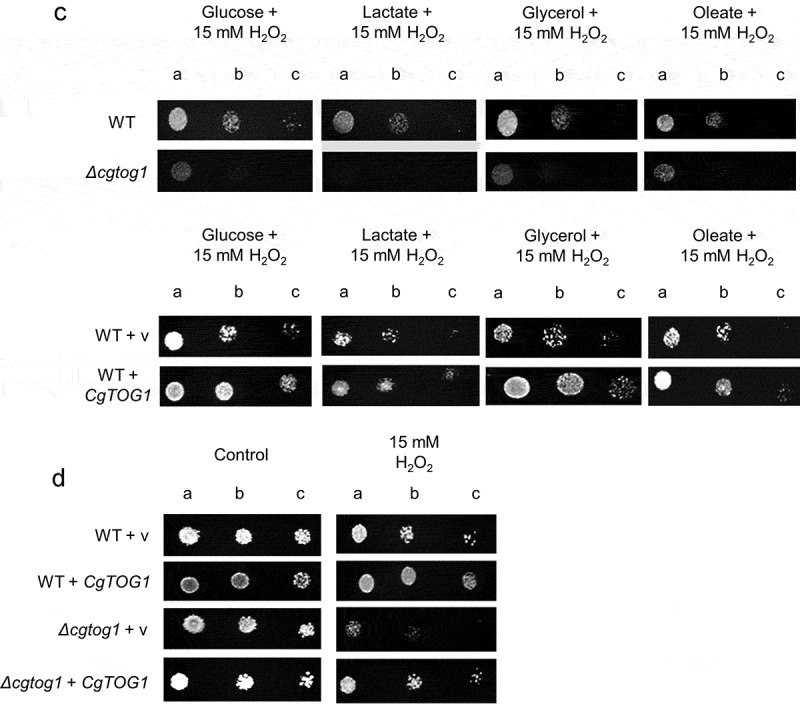
(Continued)

Since the use of alternative carbon sources and oxidative stress represent concurrent environmental stresses felt by microbial pathogens in the phagosome, we investigated a possible role of CgTog1 in these conditions. The *Δcgtog1* strain displays increased susceptibility when compared to the wild type strain, whereas *CgTOG1* overexpression confers a fitness advantage (Figure 1c). We could not clearly see an effect of *CgTOG1* deletion in the susceptibility to oxidative stress in cells growing in alternative carbon sources, when compared to oxidative stress alone, except eventually for lactate. These results indicate that CgTog1 may play a role in mediating *C. glabrata* response to phagosome combined antimicrobial stresses. Considering the absence of phenotypic differences when probing growth defects using alternative carbon sources alone, these results seem to indicate that resistance to H_2_O_2_ may be the most direct impact of CgTog1 expression. To further assess this, we investigated the ability of CgTog1 to rescue the susceptibility phenotype of the *Δcgtog1* mutant. Accordingly, the expression of *CgTOG1* was able to rescue the observed susceptibility phenotype to H_2_O_2_ (Figure 1d), indicating a role for this TF in oxidative stress resistance.

Altogether, this data indicates that CgTog1 mediates oxidative stress resistance in *C. glabrata*. Moreover, it suggests that the role of Tog1 has partially diverged in *C. glabrata* relative to the model yeast *S. cerevisiae*, which may be the result of co-evolution with the human host.

### CgTog1 contributes to the reduce the intracellular concentration of ROS during oxidative stress

**Figure 2.**
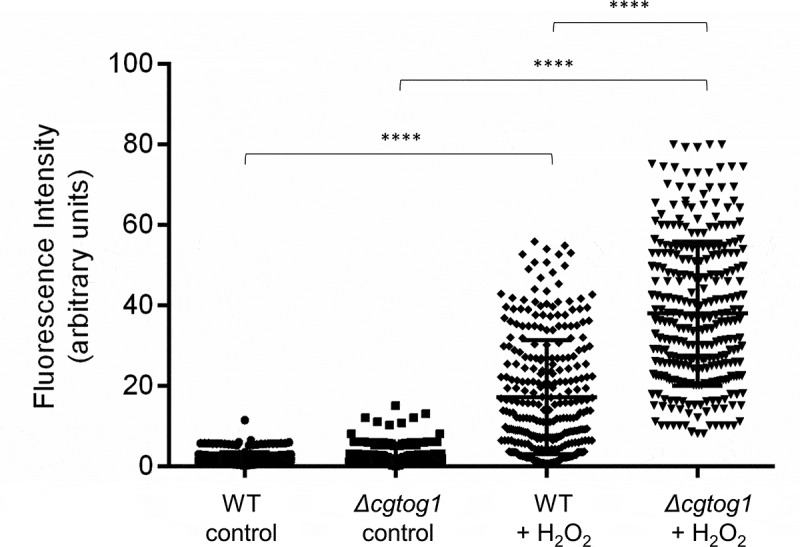
***CgTOG1* contributes to reduce the intracellular accumulation of ROS**. Comparison of intracellular ROS concentration of the KUE100 *C. glabrata* wild type and derived *Δcgtog1* deletion mutant in control conditions or upon 1 h of H_2_O_2_ stress. The estimation of intracellular ROS is based on the fluorescence intensity values exhibited by yeast cells upon incubation with the cell-permeant 2ʹ,7ʹ-dichlorodihydrofluorescein diacetate indicator H_2_DCFDA. The displayed values correspond to at least three independent experiments. Error bars represent the corresponding standard deviation

The susceptibility phenotype of the *Δcgtog1* mutant to the oxidative stress inducer H_2_O_2_ led us to investigate if this TF contributes to control the concentration of ROS in *C. glabrata* cells as part of its mode of action. The presence of intracellular ROS during H_2_O_2_ exposure was assessed using the fluorescent probe H_2_DCFDA. A significant increase in the intracellular concentration of ROS inside *C. glabrata* cells was observed after 1 h of H_2_O_2_ stress (Figure 2). An even stronger increase in intracellular ROS concentration was observed in *Δcgtog1* cells (Figure 2), indicating that *CgTOG1* is required to reduce the formation of intracellular ROS in *C. glabrata* during oxidative stress cause by H_2_O_2_.

### CgTog1 is constitutively localized in the cell nucleus

**Figure 3.**
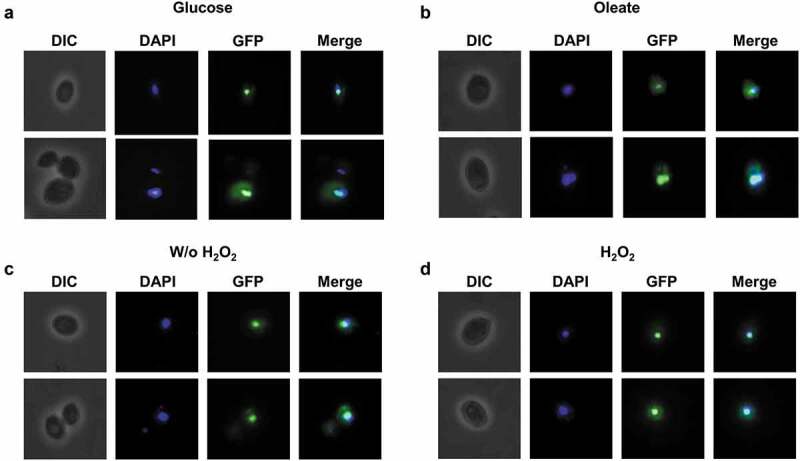
***CgTOG1* is localized to the nucleus**. Fluorescence of L5U1 *C. glabrata* cells harboring the pGREG576_MTI_*CgTOG1* plasmid after 5 h of copper-induced recombinant protein production in BM-U medium. (a,b) Cells were washed with PBS buffer and transferred to YP medium with glucose or oleate as carbon source for 1 h. (c,d) Cells were washed with PBS buffer and transferred to fresh medium with or without 15 mM H_2_O_2_ for 1 h. Results indicate that CgTog1_GFP fusion protein localized to the nucleus under all conditions tested. The displayed images are representative of at least three independent experiments

To screen for conditions that might lead to CgTog1 activation, the subcellular localization of the CgTog1_GFP fusion protein was inspected by fluorescence microscopy in medium containing oleate as an alternative carbon source and during H_2_O_2_ stress. However, the subcellular localization of CgTog1_GFP was unchanged during growth in glucose or oleate as carbon source, being always localized to the nucleus, as verified by co-localization with DAPI nuclear staining (Figure 3a,b). The same pattern was observed before and after H_2_O_2_ exposure (Figure 3c,d). Therefore, neither carbon source nor H_2_O_2_ treatment change the relative distribution of CgTog1 in *C. glabrata* cells, indicating that activation of its target genes is not based on translocation of the transcription factor to the nucleus. This observation uncovers a distinct activation mechanism from other ORS regulators, such as Yap1, which is imported to the nucleus upon oxidative stress [29].

### *Oxidative stress induces* CgCTA1 *expression in a* CgTOG1*-independent manner*

**Figure 4.**
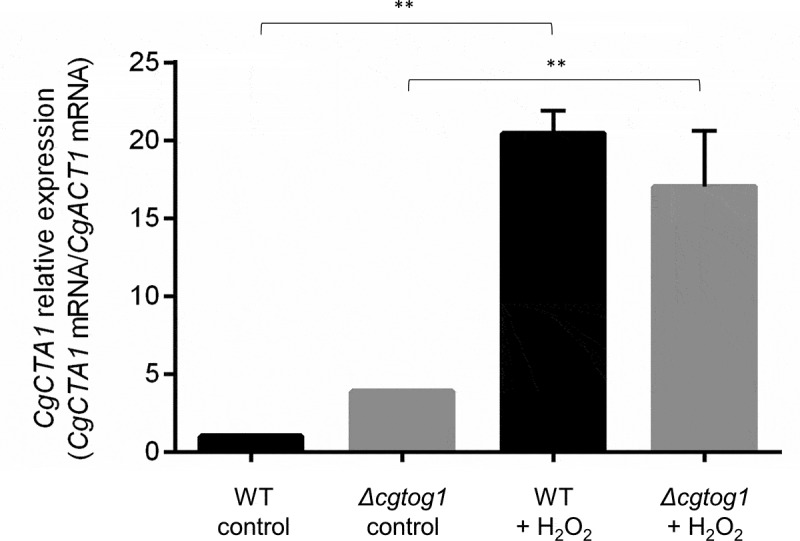
***CgCTA1* expression is independent of *CgTOG1***. Comparison of the variation of *CgCTA1* transcript levels determined by RT-PCR in the KUE100 *C. glabrata* wild type and derived *Δcgtog1* deletion mutant in control conditions or upon 1 h of 15 mM H_2_O_2_ stress. Transcript levels of *CgACT1* were used for normalization. Expression values are the average of at least three independent experiments. Error bars represent the corresponding standard deviation

The finding that *CgTOG1* is required for *C. glabrata* oxidative stress resistance exerted by H_2_O_2_ motivated us to inquire if the catalase gene *CgCTA1* is regulated by this TF. The transcript levels of the *CgCTA1* gene were quantified by RT-PCR in the presence or absence of H_2_O_2_ and its expression in the *Δcgtog1* mutant was compared to that of the wild type parental strain. The basal expression of the catalase gene is higher in *Δcgtog1* cells when compared to wild type cells. A strong upregulation of *CgCTA1* expression (~20-fold) was observed after 1 h of H_2_O_2_ stress (Figure 4). Interestingly, the induction of catalase gene expression is affected by *CgTOG1* deletion, showing a 4-fold decrease compared to wild type induction. However, given the different basal expression levels, both wild type and *Δcgtog1* mutant strains attain similar *CgCTA1* expression levels during H_2_O_2_ stress (Figure 4).

### *Role of* CgTOG1 *in the transcriptome-wide changes occurring in response to H_2_O_2_ in* C. glabrata

The *Δcgtog1* susceptibility phenotype to H_2_O_2_ independent of catalase expression warranted further investigation into the role of this transcription factor in mediating oxidative stress resistance. To clarify the role of CgTog1 in *C. glabrata* response to H_2_O_2_, transcriptome-wide expression changes occurring upon H_2_O_2_ exposure were assessed and the participation of *CgTOG1* in the observed expression program was evaluated.

**Figure 5.**
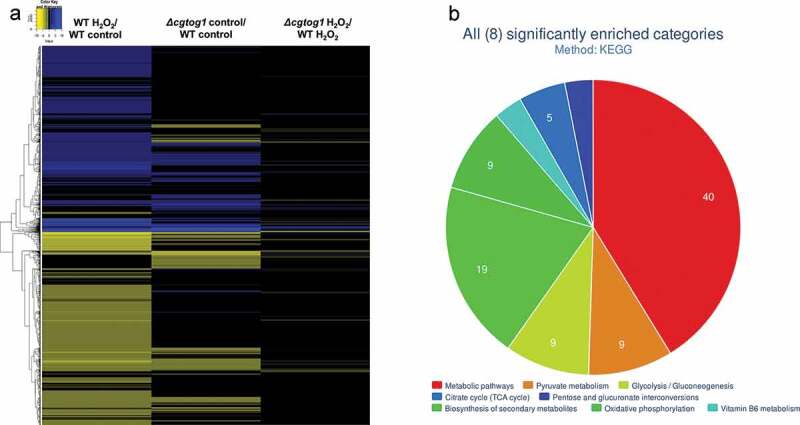
***C. glabrata* gene expression during oxidative stress and its regulation by *CgTOG1***. (a) Heatmap depicting the global transcriptomics response to H_2_O_2_ in KUE100 *C. glabrata* wild type and the derived *Δcgtog1*. (b) Systematic analysis of differentially expressed genes regulated by *CgTOG1* with the help of enriched KEGG pathways calculated with FungiFun2. The significantly enriched pathways are shown

The expression of 2558 genes was altered in *C. glabrata* cells following exposure of 1 h to 15 mM H_2_O_2_ (log_2_ fold change > 0.5 or < −0.5, adjusted p-value < 0.05) (Supplementary Table S1). These conditions appear to elicit a significant stress, translated by the pronounced transcriptional response: the upregulation of 1168 (46%) and the downregulation of 1390 (54%) genes (Figure 5a). As expected, this dataset includes the upregulation of genes involved in the core oxidative stress response: the hallmark catalase gene (*CgCTA1*), the superoxide dismutase gene (*CgSOD1*), oxidative stress resistance regulators (*CgYAP1, CgSKN7*) and several peroxidases and reductases (*CgTSA1, CgTRR1, CgTRX2, CgTRX3, CgMXR1, CgSRX1, CgGRX8, CgGRX4, CgGPX2*).

To understand the role of CgTog1 in the oxidative stress response of *C. glabrata*, the differential basal gene expression of *Δcgtog1* vs wild type cells and the differential gene expression in the presence of H_2_O_2_ was further evaluated. Deletion of CgTog1 was found to deregulate the basal expression of 607 genes, resulting in increased expression of 233 genes and reduced expression of 374 genes. CgTog1 was found to control the basal expression of antioxidant genes and redox processes (*CgGPX2, CgIDP1, CgTSA1, CgERV1, CgPAN5*). Interestingly, CgTog1 was found to significantly contribute to the basal expression of protein folding/proteasome homeostasis genes (*CgPRE1/3/4/5/7, CgRPN1/5/7/11, CgPUP3*).

While assessing the impact of CgTog1 deletion during oxidative stress, 112 genes display increased expression, while 147 genes show decreased expression (Figure 5a; Supplementary Table S1). To determine the possible CgTog1-regulated genes that may mediate the oxidative stress resistance phenotype, those found to display altered expression in *Δcgtog1* cells in the presence of H_2_O_2_ but not in control conditions were analyzed. In this dataset, the expression of 115 genes was found to be activated by CgTog1 specifically under H_2_O_2_ stress, while 89 genes were found to be repressed. In accordance with our previous RT-PCR data, the catalase gene *CgCTA1* is upregulated during oxidative stress, but its final expression level is not affected by *CgTOG1* deletion. Among the activated genes, 13 were also found overexpressed in the wild type response to H_2_O_2_, which could represent the core gene set activated by CgTog1 to mediate the oxidative stress response (Table 1). This subset includes the ORF *CAGL0F00649g*, encoding a putative kinase with similarity to ScRck2, known to be involved in the oxidative stress response in *S. cerevisiae* [30]. Other than this target, the transcriptome profile of *Δcgtog1* cells did not reveal deregulation over core OSR genes, but rather mitochondrial function, redox and energy pathways (*CgADH6, CgPDC6, CgALD6, CgMRP10, CgCYB2, CgDLD1, CgCYT1*), as analyzed using FungiFun2 [31] (Figure 5b).

**Table 1.** List of CgTog1 activated genes which are also upregulated in the wild type oxidative stress response caused by H_2_O_2_. Expression values represent log_2_ fold changes

ORF/Gene Name	*S. cerevisisae* homolog	Function	WT H_2_O_2_/WT control	*Δcgtog1* H_2_O_2_/WT H_2_O_2_
*MIG1*	*MIG1*	Transcription factor involved in glucose repression	2,58	−1,79
*CAGL0F05709g*	*ATC1*	Cation stress response	2,56	−1,24
*CAGL0H02491g*	*COX7*	Subunit VII of cytochrome c oxidase	2,48	−0,68
*CAGL0F00649g*		Response to oxidative and osmotic stress	2,47	−3,63
*CAGL0I00116g*		Unknown function	2,43	−1,06
*CAGL0B02079g*	*AZR1*	Plasma membrane transporter	2,30	−1,56
*NCE103*	*NCE103*	Beta carbonic anhydrase	2,24	−1,45
*CAGL0A01606g*	*HOP2*	Meiosis-specific protein	1,93	−0,85
*CAGL0E00649g*	*PTC6*	Mitophagy	1,67	−0,88
*CAGL0D02640g*		Unknown function	1,62	−3,64
*CAGL0M12001g*		Unknown function	1,07	−1,76
*CAGL0G07931g*	*MRPS12*	Mitochondrial translation	0,86	−0,70
*CAGL0K07942g*		Unknown function	0,61	−0,66

### *The transcription factor* CgTOG1 *is required for* Candida glabrata *survival to phagocytosis*

The ability of pathogens to cope with oxidative stress in the phagosome environment is a key virulence feature that mediates phagocyte survival. The role of *CgTOG1* in conferring resistance to oxidative stress prompted us to investigate its impact in *C. glabrata* survival upon phagocytosis using the *Galleria mellonella* infection model hemocytes, the insect equivalent to mammalian lymphocytes.

**Figure 6.**
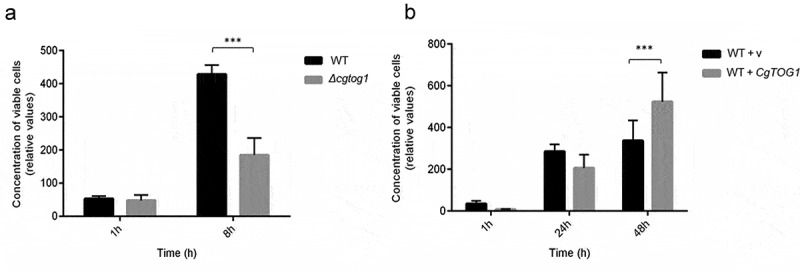
***CgTOG1* is required for survival upon phagocytosis**. (a) The concentration of viable KUE100 *C. glabrata* wild type (black bars) or derived *Δcgtog1* deletion mutant (gray bars) after co-culture with *G. mellonella* hemocytes using a MOI of 1:5. (b) The concentration of viable L5U1 *C. glabrata* wild type strain, harboring the pGREG576 cloning vector (black bars), or the pGREG576_MTI_*CgTOG1* expression plasmid (gray bars) after co-culture with *G. mellonella* hemocytes using a MOI of 1:5. The displayed results are relative to the concentration of viable cells inoculated at time zero and are the average of at least six independent experiments. Error bars represent the corresponding standard deviation

The amount of viable *C. glabrata* cells found within hemocytes was assessed up to 48 h of co-culture. After 1 h, no differences can be observed in the survival of internalized *C. glabrata* between the wild type and the *Δcgtog1* mutant strains. However, after only 8 h the population of wild type cells found within hemocytes was found to be 2.5-fold higher than that of the *Δcgtog1* mutant (Figure 6a). Consistent with the previous results, the overexpression of *CgTOG1* was found to lead to a 35% increase in cell proliferation within hemocytes at 48 h of internalization (Figure 6b). Overall, these results show that the TF CgTog1 is involved in *C. glabrata* virulence in the *G. mellonella* infection model by enabling yeast survival inside phagocytic cells.

### *The role of CgTog1 regulon in* C. glabrata *virulence*

Upon phagocytosis pathogens activate alternative carbon source pathways due to glucose starvation (e.g., glyoxylate cycle) and oxidative stress response genes [10,24,25]. Moreover, both glucose starvation and oxidative stress have been described to elicit a somewhat similar transcriptional response in *C. glabrata* [10,13,25].

**Figure 7.**
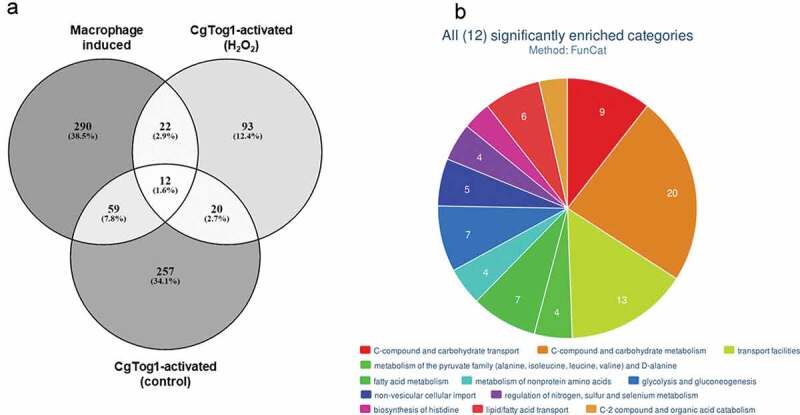
**The *CgTOG1* regulon in the transcriptomics context of macrophage engulfed cells**. (a) Number of differentially expressed genes that constitute the *CgTOG1* activated genes in either control conditions or H_2_O_2_ stress, compared to the activated transcriptional response of *C. glabrata* upon macrophage engulfment. Overlapping genes were identified based on their systematic ORF designations. (b) Systematic analysis of genes commonly activated by *CgTOG1* and upon macrophage engulfment, with the help of enriched FunCat functional categories calculated with FungiFun2. The significantly enriched pathways are shown

The role of CgTog1 in mediating hemocyte survival and oxidative stress resistance, together with its regulation of energy/metabolism genes, including players in the TCA and glyoxylate cycles (Table 1; Supplementary Table S1), led us to compare the genes activated by CgTog1 in control conditions or H_2_O_2_ stress with the described transcriptome-wide response induced in murine and human macrophage-engulfed *C. glabrata* cells [8,24], as to ascertain its relevance in this context. An overlap of 93 genes was revealed (Figure 7a, Supplementary Table S1), with the most enriched biological functions featuring: amino acids metabolism, carbon/energy, and lipid and fatty acid metabolism and transport, as analyzed by FungiFun2 [31] (Figure 7b). From the 22 genes upregulated in macrophages and activated by CgTog1 in response to H_2_O_2_, 4 were also upregulated in the wild type transcriptional response to oxidative stress: *CgMIG1, CgNCE103, CAGL0F00649g*, and *CAGL0F05709g* (*CgATC1)*.

Interestingly, a search for gene syntenic context using CGOB [32] and *ScTOG1* as query revealed no homologs in *Candida* spp. from the CTG clade (*C. albicans, C. parapsilosis, C. tropicalis*) or *C. auris*. Likewise, Blastp search for amino acid similarity to the CgTog1 protein did not reveal any unequivocal homolog (best match = E-value 10^−16^) in any of the aforementioned species. This indicates that the Tog1 TF is present in the two phylogenetically close yeasts *C. glabrata* and *S. cerevisiae* (albeit with distinct functions) but is absent in the remaining (distantly related) *Candida* species. These facts appear to support a unique function for *CgTOG1* as a virulence determinant in which is specific of *C. glabrata*.

## Discussion

In this study, the TF CgTog1 was found to be a determinant of virulence in *C. glabrata*. Unlike its *S. cerevisiae* counterpart, it is not required for growth in alternative carbon sources, but rather for oxidative stress resistance. The limited homology between the two Tog1 proteins can be a signature of its evolution toward a distinct function. The two proteins share 26% identity, which is mainly localized in the zinc-finger DNA binding domain. Both proteins harbor a fungal transcription factor regulatory middle homology region (MHR), typically present in TFs with a N-terminal GAL4-like zinc finger DNA-binding domain and has a regulatory role. Interestingly, the MHR is significantly shorter in CgTog1 (80–92 amino acids long) when compared to ScTog1 (223–249 amino acids longs). This could represent an important feature underlying the different phenotypes regulated by the two Tog1 proteins.

The apparent loss of CgTog1 function in alternative carbon source utilization and focus on oxidative stress response may possibly correlate with the evolution of *C. glabrata* in the human host, where the production of ROS is a major antimicrobial strategy inside phagocytic cells [11,12]. Indeed, *CgTOG1* is shown in this study to modulate the concentration of intracellular ROS, indicating a role either in ROS detoxification or intracellular redox balance, and to mediate *C. glabrata* survival upon phagocytosis.

Transcriptomics profiling during H_2_O_2_ exposure revealed that the response of *C. glabrata* is quite extensive, including the activation of core OSR genes with additional concurrent transcriptional patterns. Previous transcriptomics studies investigating the response of *C. glabrata* to oxidative stress have probed a lower concentration of H_2_O_2_ and a shorter exposure time [13,33]. However, while this concentration is stressful for *S. cerevisiae*, it is only mildly so for *C. glabrata* [33]. The distinct experimental conditions may justify why our study observed a larger transcriptional response (>1000 activated genes in this study versus >200 activated genes in previous studies). As expected, both approaches allowed to capture the activation of core OSR genes (*CgCTA1, CgTRR1, CgTRX2, CgTSA1, CgGPX2*), while our study also identified additional and possibly partial indirect responses. Considering the higher resistance of *C. glabrata* to oxidative stress, the use of more stressful conditions in this study presumably allowed to capture a more complete picture of its transcriptional response. Enriched groups include regulation of transcription, denoting the activation of multiple regulatory pathways (*CgHOG1, CgCAD1, CgYAP5, CgYAP7, CgHAP1*). Interestingly, response to high levels of H_2_O_2_ also encompasses increased expression of the OSR regulators *CgYAP1* and *CgSKN7*, and not only their target genes. Moreover, the comparison between the transcriptional response between H_2_O_2_ stress and phagocytosis reveals a common activation of oxidative stress genes (*CgCTA1, CgTRR1, CgTRX2, CgGRE2(B)*), which conforms to the notion that yeast cells suffer from oxidative stress upon phagocytosis. Additionally, common transcriptional responses include activation of utilization of alternative carbon sources (*CgCAT8, CgMIG1, CgPOX1*) and energy pathways such as the glyoxylate (*CgICL1*) and TCA cycles (*CgKGD1, CgSDH2, CgFUM1, CgACO1, CgSDH2*). These findings highlight an overlap in the transcriptional response to high oxidative stress and phagocytosis, mostly translated in the activation of not only OSR genes, but also key energetic pathways. This may underlie the success of *C. glabrata* as an oxidative and phagocytosis resistant pathogen.

Interestingly, *CgTOG1* regulates oxidation-reduction processes, but it does not control the expression of typical OSR genes, including the catalase gene *CgCTA1*. Validation of *CgCTA1* expression by RT-PCR was performed and revealed an apparent upregulation of the catalase gene when *CgTOG1* is deleted. This may indicate a role in *CgCTA1* basal expression regulation. However, no regulation of catalase gene expression by CgTog1 under H_2_O_2_ stress was found in the transcriptomics results. This data was validated by RT-PCR, which shows a reduction in *CgCTA1* induction in the absence of the TF, but to levels similar to those attained in the wild type strain.

By analyzing the regulation of *CgCTA1* using the PathoYeastract database [34,35], its expression is activated by four TFs, including the OSR regulators *CgYAP1* and *CgSKN7. CgTOG1* is not known to be part of the regulons of either *CgYAP1* or *CgSKN7*, nor it regulates either of them as determined by our data, indicating that its mode of action is outside of the traditional OSR network. In fact, it exerts transcriptional regulation that mimics the hallmark of macrophage-internalized pathogens: reprogrammed carbon metabolism based on increased nucleogenesis, glyoxylate cycle and fatty acid degradation pathways [23]. It is striking that a determinant of OSR presents such a regulatory portrait, even during H_2_O_2_ stress. These observations appear to be consistent with studies in *S. cerevisiae* that show respiration disruption is correlated with H_2_O_2_ sensitivity [36,37], while reduced cofactors (e.g., NADPH) are especially relevant in oxidative stress conditions, as NADPH is a final electron donor in glutathione and thioredoxin systems [38]. This data is further corroborated by other *C. glabrata* studies reporting mitochondrial processes and activity of dehydrogenases as crucial under oxidative stress [13].

Upon phagocytosis, reprogramming of nutrient metabolic pathways, particularly carbon and nitrogen biosynthesis, takes place due to nutrient limitation [8,14,24]. Our transcriptomics analysis identified *CgTOG1* as a regulator of these central responses to phagosome stress, which is concordant with its *G. mellonella* hemocyte survival phenotype and the overlap between activated genes in both glucose starvation and OSR in *C. glabrata* [13]. Previous studies reported the upregulation of gluconeogenesis, glyoxylate and methylcitrate cycles and oxidation of fatty acids upon *C. glabrata* phagocytosis by murine or human macrophages [8,24]. In this study, *CgTOG1* was found to contribute to the activation of these pathways, including previously identified genes in such responses, such as *CgMLS1* (glyoxylate cycle), *CgICL2* and *CgPDH1* (methylcitrate cyle), *CgFBP1* and *CgPCK1* (gluconeogenesis). Interestingly, *CgMDH2*, encoding an enzyme involved in the glyoxylate cycle and gluconeogenesis that interacts with Pck1 and Fbp1 in *S. cerevisiae* [39], is also regulated by CgTog1. The genes *CgIDP2, CgACS1* and *CgICL2*, which are activated upon non-fermentable carbon source growth in *S. cerevisiae* [40–42], are also regulated by CgTog1. Furthermore, the glucose limitation response TF *CgMIG1* was found to be induced by *CgTOG1* and in the wild type response to H_2_O_2_, being induced in macrophages as well, which hints for another regulatory network involved in *C. glabrata* virulence. Other than regulation over central carbon and energy pathways, CgTog1 also shares regulation over β-oxidation of fatty acids and fatty acid metabolism genes that are activated during phagocytosis, including *CgYAT1, CgYAT2, CgCAT2, CgCRC1, CgYMC2, CgFAA2* and *CgECI1*. Fatty acid β-oxidation is recognized as a possible major energy generation pathway in phagocytosed *Candida* cells [24,25,43]. The regulation of fatty acid metabolism by CgTog1 may therefore represent an additional basis for the hemocyte survival phenotype mediated by *CgTOG1*.

Several of the genes concurrently found to be activated during phagocytosis and by CgTog1 are involved in mitochondrial functions and peroxisomal activity, with potential impact on the redox state of the cells (e.g., fatty acid oxidation). This indicates a possible role for CgTog1 in the key regulation of central carbon metabolism and energy pathways, although not required for growth on alternative carbon sources. This would be consistent with the role of CgTog1 in mediating hemocyte survival: specific pathways would be activated for the uptake of alternative carbon sources with a concurrent role of CgTog1 in prompting central carbon metabolism reshuffling through, for instance, the glyoxylate cycle. Such a role would correlate well with control over the cells redox state via regulation of oxidative phosphorylation and fatty acid oxidation, which in turn can impact resistance to oxidative stress. In conjunction, adaptive energy metabolism and oxidative state are the main drivers of pathogen survival upon phagocytosis and the CgTog1 is found as a central piece in this response.

Differential regulation over nitrogen metabolism pathways is also a feature of *CgTOG1*. Previous studies have reported the upregulation of arginine and lysine biosynthetic pathways [24], while our data also indicates an enrichment in the histidine pathway. Consistently, our results also highlight an enrichment in amino acid transport activity, including the previously observed general amino acid permease *CgGAP1* [24]. The conclusions drawn from this study reinforce the importance of multi stress resistance for prevalence within the host. Importantly, this TF is not only functionally distinct from its counterpart in the nonpathogenic *S. cerevisiae*, but it is absent from other clinically relevant *Candida spp*., including *C. albicans, C. parapsilosis, C. tropicalis* and *C. auris*. It is plausible that distinct evolutionary paths have occurred for *C. glabrata* to retain this TF, while its evolution with the human host may have contributed for CgTog1 to have become distinct from its *S. cerevisiae* counterpart.

Overall, this study describes a new regulator of virulence in *C. glabrata*. Our results show that the CgTog1 TF mediates phagocyte survival by limiting the accumulation of intracellular ROS and a concerted regulation over OSR, nutrient limitation response and energy metabolism. This transcriptional pattern constitutes the hallmark of pathogen survival upon phagocytosis and the prevalent role of *CgTOG1* in this mechanism has been confirmed by the activation of common genes during oxidative stress and upon *C. glabrata* phagocytosis. Together with the transcriptomics data showing a role for *CgTOG1* in the transcriptional response to phagocytosis, a prominent function in *C. glabrata* virulence is proposed. We present evidence for a specific regulator of *C. glabrata* survival in immune cells, which could add another piece to the puzzle on how this species is capable of not only survive but replicate inside phagocytes.

## Materials and methods

### Strains, plasmids and growth media

*Candida glabrata* parental strain KUE100 [44] and derived single deletion mutants were batch-cultured at 30ºC, with orbital agitation (250 rpm) in basal medium (BM) or Yeast extract-Peptone-Dextrose (YPD) medium. BM has the following composition (per liter): 1.7 g yeast nitrogen base without amino acids or NH_4_^+^ (Difco), 20 g glucose (Merck) and 2.65 g (NH_4_)_2_SO_4_ (Merck). YPD has the following composition (per liter): 20 g glucose (Merck), 20 g Peptone (Merck) and 10 g Yeast extract (Merck). *C. glabrata* strain L5U1 (*cgura3Δ0, cgleu2Δ0*) [19], kindly provided by John Bennett from the National Institute of Allergy and Infectious Diseases, NIH, Bethesda, USA, was grown in BM supplemented with 20 mg/L uracil and 60 mg/L leucine. L5U1 strains harboring pGREG576 derived plasmids were grown in the same medium lacking uracil. KUE100::URA- and the derived KUE100_*Δcgtog1*:URA- strains, constructed in this study, harboring pGREG576 derived plasmids were grown in BM. Solid media contained, besides the above-indicated ingredients, 20 g/L agar (Iberagar). The plasmid pGREG576 was obtained from the Drag&Drop collection [45]. The plasmid pV1382 [46] was obtained from Addgene (Addgene plasmid# 111436).

### *Disruption of* C. glabrata CgTOG1 *(ORF* CAGL0F09229g)

The deletion of the *C. glabrata* gene encoded by ORF *CAGL0F09229g*, addressed in this study, was carried out in the parental strain KUE100, using the method described by Ueno *et al*. [47]. Genes of interest were replaced by a DNA cassette including the *CgHIS3* gene, through homologous recombination. The PCR primers used to generate the replacement cassette for each gene and the primers used for PCR confirmation of gene deletion are present in Supplementary Table S2. The pHIS906 plasmid including *CgHIS3* was used as a template and transformation was performed as described previously [44].

### *Cloning of the* C. glabrata CgTOG1 *(ORF* CAGL0F09229g)

The pGREG576 plasmid from the Drag&Drop collection was used as described before to clone and express the *C. glabrata CgTOG1* gene [48–53]. pGREG576 was acquired from Euroscarf and contains a galactose inducible promoter (*GAL1*), the yeast selectable marker *URA3* and the *GFP* gene, encoding a Green Fluorescent Protein (GFPS65T), which allows monitoring of the expression and subcellular localization of the cloned fusion protein. *CgTOG1* DNA was generated by PCR, using genomic DNA extracted from the sequenced CBS138 *C. glabrata* strain. The designed primers contain, besides a region with homology to the first and last 22 nucleotides of the *CgTOG1* coding region (italic), nucleotide sequences with homology to the cloning site flanking regions of the pGREG576 vector (underlined). The amplified fragments were co-transformed into the parental *S. cerevisiae* strain BY4741 with the pGREG576 vector, previously cut with the restriction enzyme SalI (NZYTech), to obtain the pGREG576_*CgTOG1* plasmid. Since the *GAL1* promoter only allows a slight expression of downstream genes in *C. glabrata*, to visualize by fluorescence microscopy the subcellular localization of the CgTog1 protein in *C. glabrata*, new constructs were obtained. The *GAL1* promoter present in the pGREG576_*CgTOG1* plasmid was replaced by the copper-inducible *MTI C. glabrata* promoter [54], giving rise to the pGREG576_MTI_*CgTOG1* plasmid. The *MTI* promoter DNA was generated by PCR, using genomic DNA extracted from the sequenced CBS138 *C. glabrata* strain. The designed primers contain, besides a region with homology to 27 nucleotides in the beginning and the last 27 nucleotides of the 1000 bp upstream region of the *MTI* coding sequence (italic), nucleotide sequences with homology to the cloning site flanking regions of the pGREG576 vector (underlined). The amplified fragment was co-transformed into the parental strain BY4741 with the pGREG576_*CgTOG1* plasmid, previously cut with SacI (NZYTech) and NotI (NZYTech) restriction enzymes to remove the *GAL1* promoter, to generate the pGREG576_MTI_*CgTOG1* plasmid. The recombinant plasmids pGREG576_*CgTOG1* and pGREG576_MTI_*CgTOG1* were obtained through homologous recombination in *S. cerevisiae* and verified by DNA sequencing. All primers used are present in Supplementary Table S2.

### *Disruption of* C. glabrata CgURA3 *(ORF* CAGL0I03080g)

The disruption of the *C. glabrata URA3* gene encoded by ORF *CAGL0I03080g*, was carried out in the parental strain KUE100 and the derived *Δcgtog1* mutant, using the CRISPR-Cas9 system from Vyas *et al*. [46]. Briefly, a *CgURA3* gRNA sequence selected from the resources made available by Vyas *et al*. [46] was cloned in the pV1382 plasmid, previously linearized with the restriction enzyme BsmBI (NEB). The *CgURA3* gRNA was obtained by oligonucleotide annealing and the product ligated into the previously linearized pV1382 plasmid to obtain the pV1382_*CgURA3* vector. The construct was verified by DNA sequencing. The plasmid was transformed into the parental strain KUE100 or the derived *Δcgtog1* mutant and cells were then directly plated on 5-Fluoroorotic acid (5-FOA) to select for URA- cells. Sequential passages in nonselective medium (YPD) were performed to avoid detrimental effects of further Cas9 expression and *CgURA3* loss of function was further confirmed by the inability to grow in medium without uracil. The introduction of pGREG576 derived plasmids in the edited strains was able to rescue the growth impairment in the absence of uracil. Sequencing of the *CgURA3* gene from the selected candidates revealed the existence of frameshifts within the ORF, thus resulting in premature stop codons as it is expected from Non-Homologous End Joining (NHEJ) correction of double-strand breaks. All primers used are present in Supplementary Table S2.

### Alternative carbon source utilization assays

The ability to use alternative carbon sources of the parental strain KUE100 was compared to that of the deletion mutant KUE100_*Δcgtog1* by spot assays. The ability of *CgTOG1* gene expression to increase wild type utilization of alternative carbon sources was also examined in the URA3- L5U1 *C. glabrata* strain, using the pGREG576_MTI_*CgTOG1* centromeric plasmid. The same plasmid was used to assess the ability of *CgTOG1* to recue susceptibility to oxidative stress, examined in the KUE100::URA- and derived deletion mutant KUE100_*Δcgtog1*::URA- strains.

KUE100 *C. glabrata* and derived deletion mutant KUE100_*Δcgtog1* cell suspensions used to inoculate agar plates were mid-exponential cells grown in YPD, until culture OD_600nm_ = 0.5 ± 0.05 was reached. Cells were washed and diluted in sterile water to obtain suspensions with OD_600nm_ = 0.05 ± 0.005. These cell suspensions and subsequent dilutions (1:5; 1:25) were applied as 4 µL spots onto the surface of solid YP plates, using distinct carbon sources: 2% glucose, 0.75% lactate, 1% glycerol or 0.1% oleic acid + 0.5% Tween80. L5U1 harboring the pGREG576_MTI_*CgTOG1 C. glabrata* cell suspensions used to inoculate the agar plates were mid-exponential cells grown in BM-U supplemented with leucine and 100 µM CuSO_4_, until culture OD_600nm_ = 0.5 ± 0.05 was reached. The cells were washed and diluted in sterile water to obtain suspensions with OD_600nm_ = 0.05 ± 0.005. KUE100::URA- and the derived deletion mutant KUE100 *Δcgtog1*::URA- cell suspensions, harboring the pGREG576_MTI_*CgTOG1* plasmid, used to inoculate the agar plates, were mid-exponential cells grown in BM supplemented with 100 µM CuSO_4_, until culture OD_600nm_ = 0.5 ± 0.05 was reached. The cells were washed and diluted in sterile water to obtain suspensions with OD_600nm_ = 0.05 ± 0.005

These cell suspensions and subsequent dilutions (1:5; 1:25) were applied as 4 µL spots onto the surface of solid YP plates supplemented with 100 µM CuSO_4_ and the concentrations of alternative carbon sources previously referred.

### Oxidative stress susceptibility assays

The susceptibility of the parental strain KUE100 toward toxic concentrations of oxidative stress inducers was compared to that of the deletion mutant KUE100_*Δcgtog1* by spot assays. The ability of *CgTOG1* gene expression to increase wild type resistance to the referred conditions was also examined in the URA3- L5U1 *C. glabrata* strain, using the pGREG576_MTI_*CgTOG1* centromeric plasmid.

KUE100 *C. glabrata* and derived deletion mutant KUE100_*Δcgtog1* cell suspensions used to inoculate agar plates were mid-exponential cells grown in BM, until culture OD_600nm_ = 0.5 ± 0.05 was reached and then diluted in sterile water to obtain suspensions with OD_600nm_ = 0.05 ± 0.005. These cell suspensions and subsequent dilutions (1:5; 1:25) were applied as 4 µL spots onto the surface of solid BM, supplemented with adequate chemical stress concentrations. L5U1 harboring the pGREG576_MTI_*CgTOG1 C. glabrata* cell suspensions used to inoculate the agar plates were mid-exponential cells grown in BM-U supplemented with leucine and 100 µM CuSO_4_, until culture OD_600nm_ = 0.5 ± 0.05 was reached and then diluted in sterile water to obtain suspensions with OD_600nm_ = 0.05 ± 0.005. These cell suspensions and subsequent dilutions (1:5; 1:25) were applied as 4 µL spots onto the surface of solid BM-U supplemented with leucine, 100 µM CuSO_4_ and the concentrations of oxidative stress inducers previously referred.

### Intracellular ROS quantification

The presence of intracellular ROS was assessed by the cell-permeant 2ʹ,7ʹ-dichlorodihydrofluorescein diacetate indicator (H_2_DCFDA; 25 mg/mL in DMSO, Santa Cruz Biotechnology, Inc.). *C. glabrata* cell suspensions from strains KUE100 and KUE100_*∆cgtog1* were prepared in BM until a standard culture OD_600nm_ = 0.5 ± 0.05 was reached and transferred to the same medium with or without 15 mM H_2_O_2_. After 1 h of incubation, cells were centrifuged (17,500 × g for 5 minutes), washed twice and resuspended in PBS buffer to a final 10^7^ cells/mL aliquots. H_2_DCFDA was added to 1 mL of 4 × 10^7^ cells/mL to a final concentration of 0.2 µL/mL and cell suspensions were incubated in the dark with orbital agitation (20 minutes, 250 rpm). Cells exposed to H_2_DCFDA were centrifuged (17,500 × g for 5 minutes), washed twice and resuspended in 1 mL PBS buffer. H_2_DCFDA-fluorescence was detected by fluorescence microscopy in a Zeiss Axioplan microscope (Carl Zeiss MicroImaging), using excitation and emission wavelength of 395 and 509 nm, respectively. Fluorescence images were captured using a cooled Zeiss Axiocam 503 color (Carl Zeiss Microscopy) and the images were analyzed with the ZEN lite software from ZEISS microscopy. Cell-to-cell fluorescence intensity was defined as the average of pixel by pixel intensity in the selected region of interest and a minimum of 100 cells/experiment were used.

### CgTog1 subcellular localization assessment

The subcellular localization of the CgTog1 protein was determined based on the observation of L5U1 *C. glabrata* cells transformed with the pGREG576_MTI_*CgTOG1* plasmid. These cells express the CgTog1_GFP fusion protein, whose localization may be determined using fluorescence microscopy. For experiments with distinct carbon sources, *C. glabrata* cell suspensions were prepared in BM-U supplemented with leucine and 100 µM CuSO_4_, until a standard culture OD_600nm_ = 0.5 ± 0.05 was reached and transferred to YPD medium or YP medium with 0.1% oleic acid + 0.5% Tween80. For experiments with H_2_O_2_ stress, cell suspensions were prepared in BM-U supplemented with leucine and 100 µM CuSO_4_, until a standard culture OD_600nm_ = 0.5 ± 0.05 was reached and transferred to fresh medium with or without 15 mM H_2_O_2_. After 1 h of incubation cells were washed twice and resuspended in 500 µL PBS buffer to which 2 µL of a DAPI solution (1 mg/mL in DMSO) was added. Cell suspensions were incubated in the dark with orbital agitation (30 minutes, 250 rpm) and centrifuged (17,500xg for 5 minutes), washed twice and resuspended in PBS buffer. The distribution of CgTog1_GFP fusion protein in *C. glabrata* living cells was detected by fluorescence microscopy in a Zeiss Axioplan microscope (Carl Zeiss MicroImaging), using excitation and emission wavelength of 395 and 509 nm (GFP) or 358 and 461 nm (DAPI), respectively. Fluorescence images were captured using a cooled Zeiss Axiocam 503 color (Carl Zeiss Microscopy).

### Gene expression measurement by RT-PCR

The transcript levels of the *CgCTA1* or *CgTOG1* genes were determined by quantitative real-time PCR (RT-PCR). For *CgCTA1* expression, KUE100 and KUE100_*Δcgtog1* strains were grown in BM; for *CgTOG1* expression, L5U1 strains harboring the pGREG576 cloning vector or the pGREG576_MTI_*CgTOG1* expression plasmid were grown in BM-U supplemented with leucine and 100 µM CuSO_4_. Cells were grown until mid-exponential phase and transferred to fresh medium (control) or fresh medium containing 15 mM H_2_O_2_ and harvested after 1 h incubation. Samples were immediately frozen at −80ºC until RNA extraction. RT-PCR was performed as described elsewhere [51–53]. Synthesis of cDNA for real time RT-PCR experiments, from total RNA samples, was performed using the Multiscribe^TM^ reverse transcriptase kit (Applied Biosystems) and the 7500 RT-PCR Thermal Cycler Block (Applied Biosystems), following the manufacturer’s instructions. The quantity of cDNA for the following reactions was kept around 10 ng. The subsequent RT-PCR step was carried out using SYBR® Green (NZYTech) reagents with default parameters established by the manufacturer and the primers in Supplementary Table S2. The *CgACT1* gene transcript levels were used as an internal reference.

### Total RNA extraction

*C. glabrata* strains KUE100 and KUE100_*∆cgtog1* were grown in BM until mid-exponential phase. Subsequently, cells were transferred to fresh medium (control) or fresh medium containing 15 mM H_2_O_2_ and were harvested after 1 h of incubation. Total RNA was isolated using an Ambion Ribopure-Yeast RNA kit, according to manufacturer’s instructions.

### Library preparation

Prior to RNA-seq analysis quality control measures were implemented. Concentration of RNA was ascertained via fluorometric analysis on a Thermo Fisher Qubit fluorometer. Overall quality of RNA was verified using an Agilent Tapestation instrument. Following initial QC steps sequencing libraries were generated using the Illumina Truseq Stranded Total RNA library prep kit with ribosomal depletion via RiboZero Gold according to the manufacturer’s protocol. Briefly, ribosomal RNA was depleted via pull down with bead-bound ribosomal-RNA complementary oligomers. The RNA molecules were then chemically fragmented and the first strand of cDNA was generated using random primers. Following RNase digestion the second strand of cDNA was generated replacing dTTP in the reaction mix with dUTP. Double stranded cDNA then underwent adenylation of 3ʹ ends following ligation of Illumina-specific adapter sequences. Subsequent PCR enrichment of ligated products further selected for those strands not incorporating dUTP, leading to strand-specific sequencing libraries. Final libraries for each sample were assayed on the Agilent Tapestation for appropriate size and quantity. These libraries were then pooled in equimolar amounts as ascertained via fluorometric analyses. Final pools were absolutely quantified using qPCR on a Roche LightCycler 480 instrument with Kapa Biosystems Illumina Library Quantification reagents.

### Gene expression analysis

Strand specific RNA-seq library preparation and sequencing was carried out as a paid service by the NGS core from Oklahoma Medical Research Foundation, Oklahoma City, Oklahoma, USA. Paired-end reads (Illumina HiSeq 3000 PE150, 2 × 150 bp, 2 Gb clean data) were obtained from KUE100 and KUE100_*∆cgtog1*. Two replicates of each sample were obtained from three independent RNA isolations, subsequently pooled together. Raw data is available at Gene Expression Omnibus (GEO) under the accession number GSE148288. Reads were trimmed using Skewer (v0.2.2) [55] and aligned to the *C. glabrata* CBS138 reference genome, obtained from the Candida Genome Database (CGD) (http://www.candidagenome.org/), using TopHat (v2.1.1) [56] with parameters -p 12 (number of threads), -g 1 (maximum amount of times a read can be mapped to the genome), – b2-very-sensitive (preset option) and – library-type fr-firststrand (account for strand specificity). HTSeq (v0.7.1) [57] was used to count mapped reads per ORF Differentially expressed genes were identified using DESeq2 [58] with an adjusted p-value threshold of 0.05 and a log2 fold change threshold of −0.5 and 0.5. Default parameters in DESeq2 were used.

### C. glabrata *phagocytosis assays in* G. mellonella *hemocytes*

*G. mellonella* hemocytes were isolated as described before [59]. Briefly, hemolymph was extracted from last instar larvae and mixed in with anticoagulant buffer (98 mM NaOH, 145 mM NaCl, 17 mM EDTA and 41 mM citric acid; pH 4.5) in a 1:1 proportion. After centrifugation at 250 *g* for 10 min and 4ºC, hemocytes were recovered and wash twice with PBS. Hemocytes pellet was suspended in Grace Insect Medium (GIM) (Thermo Fisher Scientific) supplemented with 10% (v/v) fetal bovine serum, 1% (w/v) glutamine, and 1% (w/v) antibiotic/antimycotic solution (10,000 units penicillin G, 10 mg Streptomycin, 25 mg/l amphotericin B), viable cells were counted with a hemocytometer and incubated at 25ºC in 24-well plates with a concentration of 2 × 10^5^ cell/ml, one day prior to be used.

Cultures of *C. glabrata* cells were grown until mid-exponential phase (OD_600nm_ = 0.4–0.6) and the appropriate volume was collected to have 7 × 10^2^ cells/ml in PBS. *Galleria* hemocyte monolayer medium was replaced with GIM without antimycotics, and then cells were infected with the yeast suspensions with a multiplicity of infection (MOI) of 1:5. After 1 h of infection at 37°C, the hemocytes were carefully washed twice with cell culture medium and fresh GIM medium was added. Viable intracellular yeast cells were measured after 1, 8, 24 or 48 h of infection, upon hemocyte lysis with 0.5%(v/v) Triton X-100 for 20 min and CFUs were counted by performing serial dilutions and plating in YPD agar plates.

### Statistical analysis

All experiments represent the average of three or more independent experiments. Error bars represent the standard deviation. Statistical analyses were performed using one-way ANOVA with Tukey’s correction. Significance levels are attributed as follows: *p < 0.05, **p < 0.01, ***p < 0.001, ****p < 0.0001.

## Supplementary Material

Supplemental Material
