# Supplementary material for: A new regulator in the crossroads of oxidative stress resistance and virulence in Candida glabrata: The transcription factor CgTog1
Source: Virulence. 2020 Oct 31;11(1):1522–38. doi: 10.1080/21505594.2020.1839231 (PMC7605352; doi:10.1080/21505594.2020.1839231)
Supplement: Supplemental Material [file KVIR_A_1839231_SM7285.zip › Supplementary Material.docx]

**Supplementary Material**

Supplementary Table S1 – List of differentially expressed genes in the distinct experimental conditions. List of genes activated during macrophage engulfment compared with the CgTog1 activated regulon.

Supplementary Table S2 – List of primers used in this study.

Supplementary Figure 1 – *CgTOG1* is overexpressed in *C. glabrata* cells harboring the pGREG576_MTI_*CgTOG1* plasmid. Comparison of the variation of *CgTOG1* transcript levels, determined by RT-PCR, in *C. glabrata* cells harboring the pGREG576 cloning vector or the pGREG576_MTI_*CgTOG1* expression plasmid in control conditions or upon 1h of 15mM H_2_O_2_ stress. Transcript levels of *CgACT1* were used for normalization. Expression values are the average of three independent experiments. Error bars represent the correspondent standard deviation.
