# Supplementary material for: A new regulator in the crossroads of oxidative stress resistance and virulence in Candida glabrata: The transcription factor CgTog1
Source: Virulence. 2020 Oct 31;11(1):1522–38. doi: 10.1080/21505594.2020.1839231 (PMC7605352; doi:10.1080/21505594.2020.1839231)
Supplement: Supplemental Material [file KVIR_A_1839231_SM7285.zip › Supplementary_Table_S2.docx]

Supplementary Table 2 – List of primers used in this study.

| **Primer** | **Sequence** |
| --- | --- |
| Deletion of *CgTOG1* | 5’-CTCCTACGAACAATTTCTGATTTTAAAAAGTGGTACAATAAACCGGTTATACCCACGGCCGCTGATCACG-3’  5’-TAAATTAATTGCCTAATATAGATATAATCCTTACTGTAAAGCCTCTAGCGACTTACCATCGTGAGGCTGG-3’ |
| Confirmation of *CgTOG1* deletion | 5’-CTCGCGAAGAAAGCCAAA-3’  5’CCGAAGTATGGGGTAATG-3’ |
| Cloning of *CgTOG1* in pGREG576 | 5’-GAATTCGATATCAAGCTTATCGATACCGTCGACA*ATGGCACCACCACTCGCGAAGA*-3’  5’-GCGTGACATAACTAATTACATGACTCGAGGTCGAC*TCATAAAGACGGGTTGTTCATA*-3’ |
| Cloning of *CgMTI* promoter in pGREG576_*CgTOG1* | 5’- TTAACCCTCACTAAAGGGAACAAAAGCTGGAGCTC*CTGTACGACACGCATCATGTGGCAATC*-3’  5’- GAAAAGTTCTTCTCCTTTACTCATACTAGTGCGGC*TGTGTTTGTTTTTGTATGTGTTTGTTG*-3’ |
| *CgACT1* gene expression | 5′-AGAGCCGTCTTCCCTTCCAT-3′  5′-TTGACCCATACCGACCATGA-3 |
| *CgCTA1* gene expression | 5’-ATGCTCACCGTTACAGATTGG-3’  5’-TGTTGGAAGCGTAGTAGTTTGG-3’ |
| *CgTOG1* gene expression | 5’-CTCGCGAAGAAAGCCAAACT-3’  5’-GCACTTTTGTACAGCAGCCT-3’ |
| Cloning of *CgURA3* gRNA in pV1382 | 5’-GATCGACCGGCCAAGGTATCGTCACG-3’  5’-AAAACGTGACGATACCTTGGCCGGTC-3’ |
| *CgURA3* amplification and sequencing | 5’-ATGTCCAGTGCCTCATATTTAC-3’  5’-GATGATGTTGCTAGGTATGATC-3’ |
