# Supplementary figures and images for: A new regulator in the crossroads of oxidative stress resistance and virulence in Candida glabrata: The transcription factor CgTog1
Source: Virulence. 2020 Oct 31;11(1):1522–38. doi: 10.1080/21505594.2020.1839231 (PMC7605352; doi:10.1080/21505594.2020.1839231)

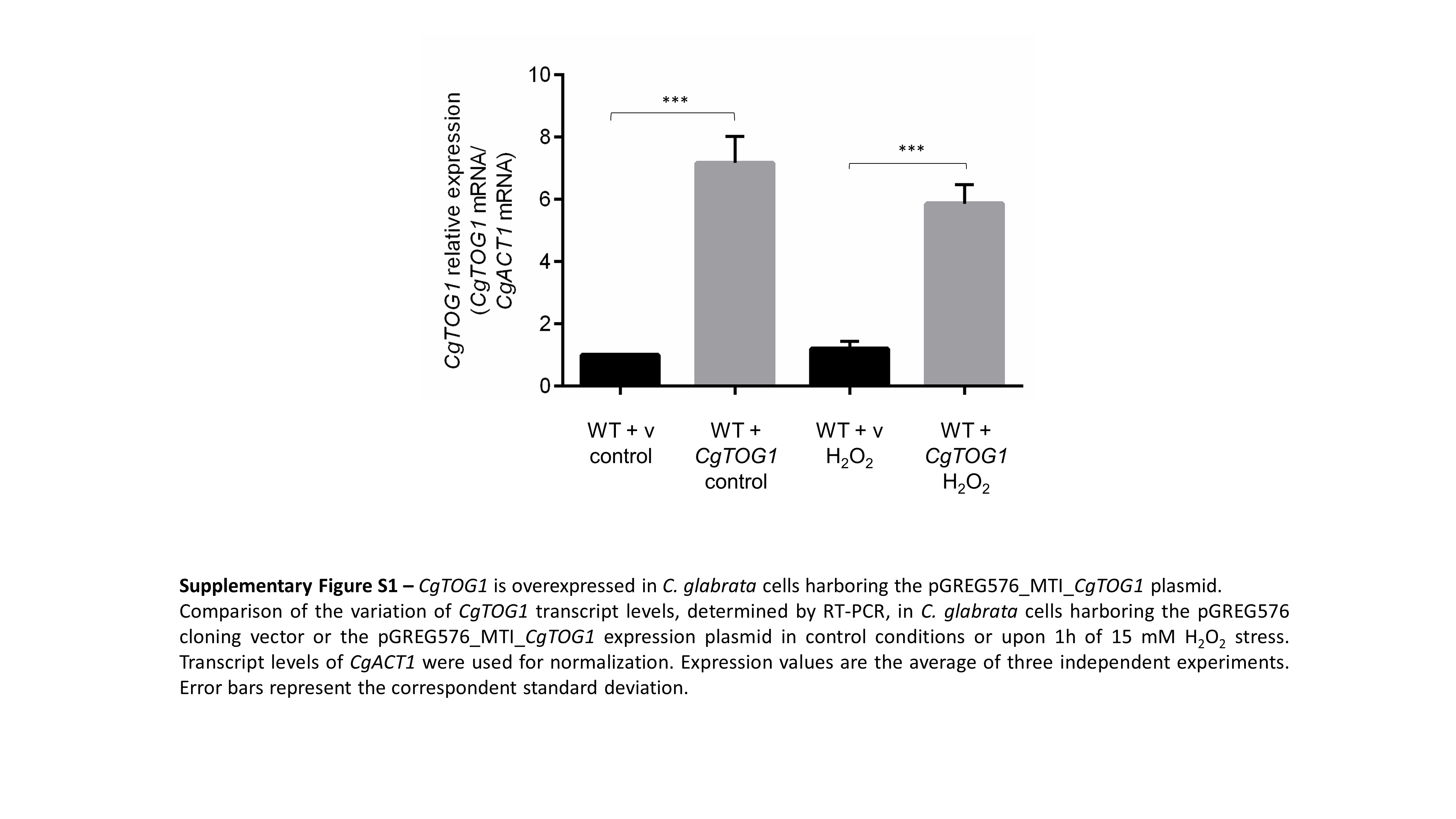

Supplement: Supplemental Material [file KVIR_A_1839231_SM7285.zip › FigS1.TIF]
